# The drive-wise project: driving simulator training increases real driving performance in healthy older drivers

**DOI:** 10.3389/fnagi.2014.00085

**Published:** 2014-05-13

**Authors:** Gianclaudio Casutt, Nathan Theill, Mike Martin, Martin Keller, Lutz Jäncke

**Affiliations:** ^1^Division of Neuropsychology, Department of Psychology, University of ZurichZurich, Switzerland; ^2^Division of Gerontopsychology, Department of Psychology, University of ZurichZurich, Switzerland; ^3^International Normal Aging and Plasticity Research Centre ZurichZurich, Schwitzerland; ^4^Division of Psychiatry Research, University of ZurichZurich, Switzerland; ^5^University Research Priority Program “Dynamics of Healthy Aging,” University of ZurichZurich, Switzerland; ^6^Center for Gerontology, University of ZurichZurich, Switzerland; ^7^Department of Neurology, Rehabilitation Centre ValensValens, Switzerland; ^8^Department of Special Education, King Abdulaziz UniversityJeddah, Kingdom of Saudi Arabia

**Keywords:** cognitive training, training effects, driving simulator, on-road driving performance, cognitive performance

## Abstract

**Background:** Age-related cognitive decline is often associated with unsafe driving behavior. We hypothesized that 10 active training sessions in a driving simulator increase cognitive and on-road driving performance. In addition, driving simulator training should outperform cognitive training.

**Methods:** Ninety-one healthy active drivers (62–87 years) were randomly assigned to one of three groups: (1) a driving simulator training group, (2) an attention training group (vigilance and selective attention), or (3) a control group. The main outcome variables were on-road driving and cognitive performance. Seventy-seven participants (85%) completed the training and were included in the analyses. Training gains were analyzed using a multiple regression analysis with planned orthogonal comparisons.

**Results:** The driving simulator-training group showed an improvement in on-road driving performance compared to the attention-training group. In addition, both training groups increased cognitive performance compared to the control group.

**Conclusion:** Driving simulator training offers the potential to enhance driving skills in older drivers. Compared to the attention training, the simulator training seems to be a more powerful program for increasing older drivers' safety on the road.

## Introduction

Due to the changing age structure in industrial countries more and more older drivers (>65 years) will drive a car on public roads either for the sake of mobility, leisure activities, or business reasons. However, there is ample evidence that on average driving performance declines and crash risks increases with increasing age (Lyman et al., 2002; Casutt et al., 2013). This decline in driving performance is also associated with a decline in perception (sensory functions), cognition (perceptual speed, higher order cognitive functions) and physiological functions as well as medical conditions (Anstey et al., 2005). Many driving errors result as a consequence of a reduction in cognitive performance, which however should be improved by training and practice (Anstey and Wood, 2011). Thus, there is growing interest in many countries to cope with increasing crash risks and decreasing driving performance in older drivers (OECD, 2001). Many strategies have been proposed so far to reduce age-related crash risks comprising specific educational programs (Stalvey and Owsley, 2003; Owsley et al., 2004; Baldock et al., 2008), withdrawal of the driving license at a particular age (Langford et al., 2004), or training cognitive functions supposed to underlie driving performance (Roenker et al., 2003; Edwards et al., 2009a,b; Ball et al., 2011).

Cognitive training regimes in older adults have consistently demonstrated improvements in the trained cognitive tasks (e.g., Karbach and Kray, 2009; von Bastian et al., 2013). However, most of these studies demonstrated transfer effects only to very similar tasks (near transfer) (Lustig et al., 2009), and virtually no transfer to other domains (far transfer; Lustig et al., 2009; Zelinski, 2009). However, the complexity of cognitive training approaches seems to be an important variable influencing far transfer of cognitive training. In fact, several studies showed that the complexity of the cognitive training increases far transfer to other cognitive domains (Basak et al., 2008; Karbach and Kray, 2009; Marmeleira et al., 2009) most likely because several cognitive functions are simultaneously trained.

In line with these findings and focusing on driving problems in older subjects there is evidence that training of particular cognitive functions can exert beneficial effects on the driving behavior. Cassavaugh and Kramer (2009) found in their driving simulator study that cognitive performance was associated with driving simulator performance. Furthermore, practicing several cognitive functions (including sensorimotor control, selective attention, working memory, and dual tasking) for eight sessions across several days resulted in improved driving performance (lane change, distance from the vehicle ahead, less driving errors, shorter reaction time). A further set of studies explored the effects of a “speed of processing training” on driving performance and identified improved performance in driving-related functions like UFOV (useful field of view), driving safety (Roenker et al., 2003), or reduced number of crashes (Ball et al., 2011) as well as number of self-reported driving difficulties (Edwards et al., 2009a,b). Interestingly, in some studies the cognitive training regimes resulted in long-lasting beneficial influences on driving behavior. For example, in the Roenker et al. (2003) study positive effects have been identified 18 months after the cognitive training. Ball et al. (2011) even reported reduced number of crashes in an observation period of 5 years.

A further strategy to improve driving performance in older adults is to practice *active* driving on a driving simulator. Simulators are frequently and intensively used in the context of various transportation situations (rail, aviation, maritime transport, space travel) especially where vehicles are very expensive in relation to a simulator. Lees et al. (2010) postulated in their review that driving simulators offer important opportunities for an efficient and valid training (interactivity, complexity, simultaneous use of different domains) not only for novice drivers, but also for older drivers.

As described above cognitive training (e.g., speed of processing) positively influences driving related variables like reduction of dangerous maneuvers, driving cessation, and driving errors (Roenker et al., 2003; Edwards et al., 2009a,b; Ball et al., 2011). Additionally, several studies have been published so far using driving simulator training approaches to improve specific and accident related driving behaviors in older adults. In older drivers reduced hazard perception was associated with reduced UFOV performance (Horswill et al., 2008). Hazard perception training in a driving simulator resulted in faster anticipation of hazardous traffic situations (Horswill et al., 2010). Other driving simulator studies investigated different aspects of problematic driving behavior (e.g., visual scanning at intersections, use of mirror while overtaking). Romoser and Fisher (2009) trained older drivers visual scanning at intersections with a driving simulator. After simulator training visual scanning (secondary looks) was improved during driving in the simulator and on the road. Furthermore, after training they examined an increase in *Rey-Osterreith Complex Figure* test (ROCFT) performance, which is associated with cognitive functions like attention, planning, and working memory (executive functions). The improved performance for “secondary looks” was observed in a follow-up 2 years later (Romoser, 2013). In another driving simulator study the use of side and rear mirrors while overtaking were trained in a sample of older drivers. Following the training, frequency of blind spot inspection increased in comparison to a training group who received no feedback (Lavallière et al., 2012). Taken together different aspects of the driving performance in older drivers (e.g., visual scanning in intersection, hazard perception, use of mirror during lane change, visuo-spatial memory) can be improved with appropriate driving simulator training (Romoser and Fisher, 2009; Horswill et al., 2010; Lavallière et al., 2012). These studies have also shown that driving simulator training can positively influence very specific aspects of cognition (e.g., executive functions) (e.g., Romoser and Fisher, 2009).

While these studies have shown that driving simulator and cognitive training regimes both do have the potential to change very specific aspects of driving (and cognition) we are more interested to examine whether general driving performance differentially benefits from a driving simulator or cognitive training. The cognitive training was designed to practice cognitive functions, which have been shown to be essential for effective driving (e.g., vigilance and selective attention) (Anstey et al., 2005; Selander et al., 2011; Casutt et al., 2014). Different to the aforementioned studies we were interested to examine whether our driving simulator training improves real on-road driving in general and not behavior in specific driving situations (e.g., visual scanning in intersection, use of mirrors during lane change). Our driving simulator training approach (practicing driving through towns, on highways, rural roads with changing traffic situations etc.) was based on a practical everyday behavior. Therefore, the used scenarios were comparable to on-road driving, which is a complex behavior and needs several psychological functions (Hakamies-Blomqvist, 1994). Our training approach is similar to multi- or dual-task training approaches, which have been shown to be more effective than single-task training (Basak et al., 2008; Marmeleira et al., 2009; Anguera et al., 2013). Real driving is a highly demanding task requiring the orchestration of many psychological functions to process many information simultaneously (traffic observation, speed control, scanning for hazard events, traffic rules, car handling). If demands increase, also the likelihood of driving errors increase (Holm et al., 2009). The relation between reduced multitasking ability and unsafe driving in older drivers and the use of compensatory strategies is well known (Sheridan, 2004; Cantin et al., 2009). Therefore, our training approach for the driving simulator training was to increase the multitasking demands in a realistic way.

Since on-road driving is difficult to assess and strongly depends on local aspects (e.g., traffic density, specific population, and specific traffic rules) we used a new on-road driving test specifically designed for a major European city (Zurich in Switzerland) with dense traffic to test whether intensive driving simulator training improves real on-road driving. In addition, we were also interested to examine whether an intensive attention training of psychological functions known to be involved in controlling driving might influence real on-road driving performance. In this context we also paid attention to examine whether our driving simulator and cognitive training exert different effects on the on-road driving performance.

Based on the results of the afore-mentioned studies we hypothesize that our driving simulator training will induce stronger improvements in on-road driving than attention training since the driving simulator training needs stronger multitasking skills and seems more attractive than attention training. In addition, we hypothesize that both training regimes (driving simulator and attention training) will improve cognitive performance **and** on-road driving compared to a no-training control group.

## Materials and method

### Participants

Participants were recruited via a newspaper articles and a newspaper advertisement about the *Drive-Wise* project. A total of 244 participants indicated interest in study participation. All of them received detailed study information and a short medical condition questionnaire (driving relevant illness, e.g., all kinds of neurological and psychiatric disorders, orthopedic problems of the upper and lower extremities etc.), medication influencing driving (e.g., drug intake influencing the central nervous system), sensory impairment (e.g., visual field < 140°). In addition, the active driving status (annual driving distance, years of possession of driving license, driving context) was assessed with a questionnaire. Participants who did not drive in all common driving contexts (urban, rural, motorway) were excluded. Ninety-one participants agreed to participate in the study and fulfilled all inclusion criteria. It is worth to mention that in Switzerland, drivers older than 70 years must undergo a screening test every 2 years (medical and cognitive screening) for renewal of their drivers' license. All participants had an original valid driver license.

Participants were not financially compensated for their travel expenses or participation, but received a detailed written feedback about performance in cognition and driving after finishing their participation. Before data collection, participants were randomly allocated either to a simulator training condition (*n* = 39), a cognitive training condition (*n* = 26), or a control group (*n* = 26). However, 14 participants dropped out during data collection. Seventy-seven individuals (55 men, 71.4%) with a mean age of 72.36 ± 5.61 (range 62–87) completed the study (Table 1). The three groups were influenced differently by the dropouts. In the simulator training group seven participants (six female) dropped out due to simulator sickness (SS) and one participant stopped due to excessive experimental demands. In the cognitive training group as well as in the control group, three participants (two female per group) finished participation due to time constraints (the entire study lasted for approximately 2 years). Study information for all groups was identical except for the particular information to run the driving simulator and cognitive training. The training setting was not explained in detail. Participants in the control group were offered the simulator training sessions (according to time of training for the two training groups) after finishing their study participation (two assessment evaluations with a 5-week waiting period in between).

**Table 1:** **Demographic characteristics**.

**Variable**	***N***	**Age (years)**	**Gender**		**Years of license**	**km/year**	**Gearbox**		
		***M (SD)***	**Male**	**Female**	***M (SD)***	***M (SD)***	**A**	**M**	**A/M**
Simulator training	31	71.74 (5.18)	22	9	49.77 (5.16)	11909 (7338)	14	11	6
Cognitive training	23	72.30 (6.46)	15	8	50.21 (5.95)	8973 (6067)	11	10	2
No training	23	73.26 (5.38)	18	5	51.34 (6.85)	10934 (5079)	8	10	5

A, automatic; M, manual.

This project (*Drive-Wise*) was approved by the Cantonal Ethic-Commission of Zurich, University Hospital of Zurich (KEK-ZH-NR: 2010-0090/0). Furthermore, traffic and police departments have granted permission to conduct the on-road test assessment. The private car of participants was labeled during the on-road test. According to information by the ethics committee, participants were informed that participation would not impinge on their driving license and that they had permission to terminate the study at any time without any negative consequences.

### Experimental apparatus

**Figure 1:**
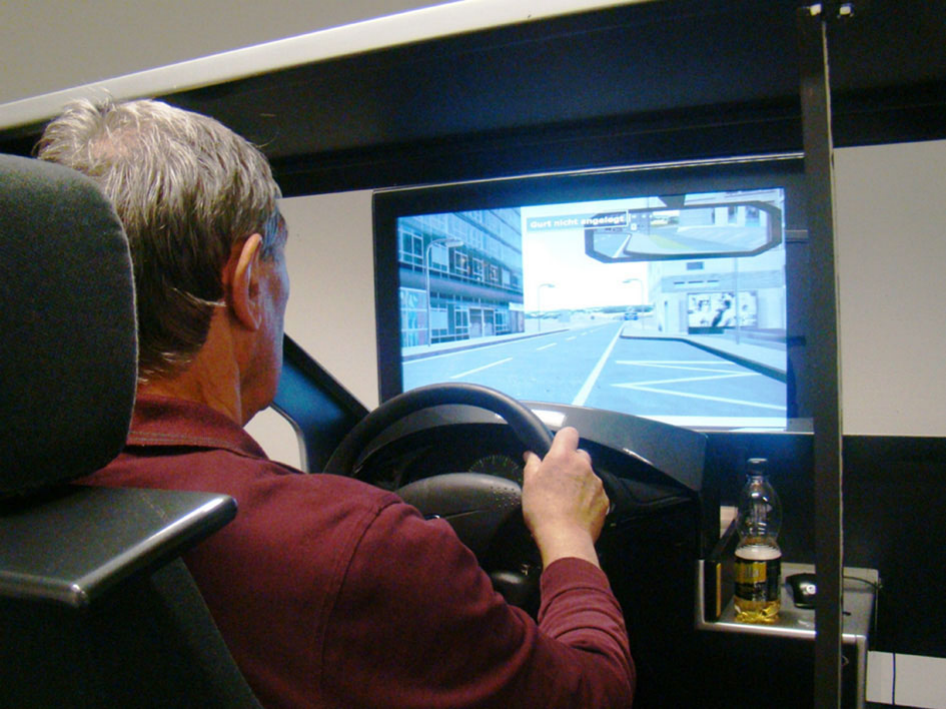
**Still photo of the used driving simulator**.

The on-road test drive was conducted in the participants' private car. Start and end point was always the department of psychology, Zurich. All tests for the cognitive test battery were conducted on a Windows Computer with a 15″ screen (resolution 1280 × 1024), distance of approximately 40 cm to the participant. Response panel and other hardware were products of Schuhfried GmbH (Schuhfried, n.d.). The training sessions of participants in the attention training group were conducted on that system as well (Phasic and tonic alertness and vigilance; CogniPlus Software from Schuhfried GmbH). Participants in the simulator training group conducted their sessions on a driving simulator type “Trainer F12PT-1L40,” software version 12 of Dr. Foerst GmbH, 32,” Samsung LCD-screen (resolution 1920 × 1080), distance of approximately 70 cm to the steering wheel (Jäncke and Klimmt, 2011). Participants sat in a driver's seat of a *Ford Focus*^©^ equipped with a steering wheel, a starter lock, a tachometer, signalers for light and blinker, wiper control switch, clutch, breaking, and throttle pedals as well as gearshift (Figure 1). The software recorded participants driving behavior. The software automatically produced traffic scenarios on a Windows 7 operating system. Moreover there were two operator screens in the same room for controlling the training sessions and giving feedback after training.

### On-road test driving assessment

The on-road test drive was conducted on public roads including district and urban streets, suburb and rural roads and a motorway passage with a total length of approximately 25 km. The test track was used as a regular basis for official on-road test exercises. A licensed driving instructor (DI) blinded to condition, sat in the front passenger seat and rated participants driving behavior directly after finishing the driving session. During the ride the instructors made notes about the driving performance, which they used for the final evaluation. The evaluation sheet (*Zurich On-road test Assessment*, ZOA), is a modified version of the formal evaluation sheet used by the DI. The DI was instructed to evaluate only cognitive aspects of driving behavior but not car handling. Seven different dimensions (Table 2) were implemented in the ZOA using six to eight items on a 5-point-scale (poor = 1, slightly insufficient = 2, sufficient = 3, good = 4, excellent = 5). An overall on-road driving performance was calculated as the mean of all on-road driving measures. This composite measure was used as dependent variable for the evaluation of on-road driving performance (Cronbach's α = 0.95). The internal reliability coefficients for each dimension ranges between 0.62–0.83.

**Table 2:** **ZOA (Zurich On-road Assessment)**.

Traffic observation (0.62)^*^	Speed at intersection (0.78)^*^	Gaze behavior (0.83)^*^	Change of direction (0.67)^*^	District dependent behavior (0.82)^*^	Use of different speed limits (0.73)^*^	Lane behavior (0.81)^*^
Motor vehicle	Intersection	Interior mirror	Turn left/right	District area	Urban streets	Straight ahead
Bicyclist	Lane change	Side view mirror	Lane change	Suburb area	Rural roads	In curves
Pedestrian	Entrance roundabouts	While start-up	Overtaking	Urban area	Knowing of speed limit	Turn left
Anticipation	Exit roundabouts	While stopping	While move along	Town area	Driving on speed limit	Turn right
Ready to slow	Entrance motorway	During lane change	Roundabouts	Rural area	Distance to cars urban streets	On light signals
Right-of-way	Exit Motorway	During overtaking	Entrance motorway	Motorway area	Distance to cars on rural road	Into account of bikes
	Acceleration	While move along	Exit motorway		Distance to cars on motorway	

* , Cronbach's α.

### Cognitive test battery

This test battery is a well-established and standardized computer-based version of the *Expert System Traffic XPSV* (Schuhfried, 2005) often used as a standard test for evaluating driving-related cognitive performance in European countries (Sommer et al., 2008, 2009, 2010). A recent paper has shown that this test battery explains 50% of the on-road test variance (Risser et al., 2008). Table 3 gives an overview of all subtests.

**Table 3:** **Expert System Traffic XPSV Schuhfried**.

**Tests**	**Cognitive paradigm**	**Output variables**	**Reliability**	**Literature**
Reaction Test (RT)	Simple choice reaction	Reaction time decision (DS) and motor speed (MS)	DS = 0.94 MS = 0.98	Schuhfried and Prieler, 1997
Cognitrone (COG)	Selective attention	Correct response, required processing time (CIAn)	0.95	Wagner and Karner, 2001
Determination Test (DT)	Complex choice reaction	Correct reactions (CR)	0.98	Schuhfried, 1998
Peripheral Perception (PP)	Field of vision, divided attention	Angular dimension (FV), tracking deviation (DA)	FV = 0.96 DA = 0.98	Schuhfried et al., 2002
Tachistoscopic Traffic Perception Test (ATAVT)	Perceptual speed	Correct response (PS)	0.80	Sommer et al., 2008, 2009
Matrices Test (AMT)	Fluid intelligence	Correct answer (FI)	0.70	Hornke et al., 2003

The *Reaction Test* (RT) is a simple choice reaction time task. From three different stimuli (yellow or red circle and an acoustic signal), participants have to discriminate the simultaneous presentation of the yellow circle and the pitched sound by pushing a corresponding target button with the right index finger as fast as possible. In all other conditions, single yellow or red circle, single pitched sound, combination of red circle and pitched sound, participants have to suppress a movement. Decision speed (DS) is measured in milliseconds by the latency from stimulus onset until lifting off the start button while the physical motor speed (MS) in milliseconds is defined as the movement time from the start button to the target button.

The *Cognitrone Test* (COG) measures selective attention. During test administration, different geometrical figures are presented block-wise. Each block comprises 60 trials. During each trial two different stimulus types are presented: four reference stimuli and one test stimulus. The four reference stimuli are presented as an array above the test stimulus. The subject's task is to decide whether the test stimulus is identical to one of the reference stimuli by pressing one of two corresponding buttons (identical vs. different). For task completion there is no time limit. Mean reaction time of correct and incongruent responses are calculated and used as a measure of selective attention (CIAn).

The *Vienna Determination Test* (DT) is used to measure reactive stress tolerance and the related reaction speed. In principle the DT requires to discriminate colors and acoustic signals, to memorize the relevant characteristics of stimulus configurations and response buttons as well as the assignment rules. In addition it is necessary to select the relevant reactions according to the assignment rules laid down in the instructions and / or learned during the course of the test. The difficulty of the DT-Test lies in the production of continuous, sustained rapid and varied reactions to rapidly changing stimuli^1^. During the 4-min test administration each subject works at the limit of his performance ability. The number of correct responses (CR) is the main variable and represents reactive stress.

The *Peripheral Perception Test* (PP) utilizes a field of vision (FV) and divided attention (DA) paradigm. Participants are sitting in front of a computer screen and perform a primary task. Beside the computer vertical panels with diodes are placed on the right and left side. The participants have to keep track of the changing diode in the periphery (secondary task) while performing the primary task. As primary task the participants have to move a cross-wire on a computer screen in order to minimize the position difference of the cross-wire with a computer-controlled moving red ball. Participants have to work simultaneously on the primary and secondary task. Every time when vertical lines appeared in the periphery they are instructed to pressing a foot pedal as fast as possible. DA is measured as the performance in the primary task (tracking deviation). FV is measured as the widest field angle at which the vertical lines of diodes are detected (during the secondary task) with respect to the distance of the screen.

The *Adaptive Tachistoscopic Traffic Perception Test* (ATAVT) is an object perception task. Photographs of traffic situations with different complexity (defined as the number of objects depicted on the photo) are presented for a short time (700–1300 ms). Participants have to decide what types of objects were presented: (1) vehicles, (2) bicycles, (3) pedestrians, (4) road signs, or (5) traffic lights. These objects are presented alone or in groups of up to five objects. The test is administered as a computerized adaptive test (CAT). The number of correctly identified objects weighted by complexity of the photograph is the dependent measure for perceptual speed (PS).

The *Adaptive Matrices Test* (AMT) is a fluid intelligence (FI) test. The stimuli are comparable to classical matrices (e.g., the Raven test). Participants have to identify the figural pattern among eight alternatives.

In previous research an overall index as a composite measure representing cognition performance has been computed on the basis of multivariate classification algorithms (artificial neural networks; NN) (Risser et al., 2008). Based on empirical evidence of Austrian and German practical on-road tests, Schuhfried GmbH categorized this composite measure into five categories. NN measures ≥4 indicate an insufficient driving behavior and also indicating that the participant would fail an on-road test. NN measures ≤3 indicate that the participant would pass the on-road test (Risser et al., 2008; Sommer et al., 2008, 2009, 2010). The NN validly estimates the composite score as demonstrated by a good jack-knife validity coefficient of *R* = 0.77. For a better data overview in the present study the scores were changed in their direction comparable to the on-road assessment measures. Therefore, scores 1 and 2 indicate that participant fail in an on-road test. Participants with a score 3 or greater would successfully complete an on-road test. This composite variable was used as dependent variable for the evaluation of cognitive performance.

### Driving simulator training

**Figure 2:**
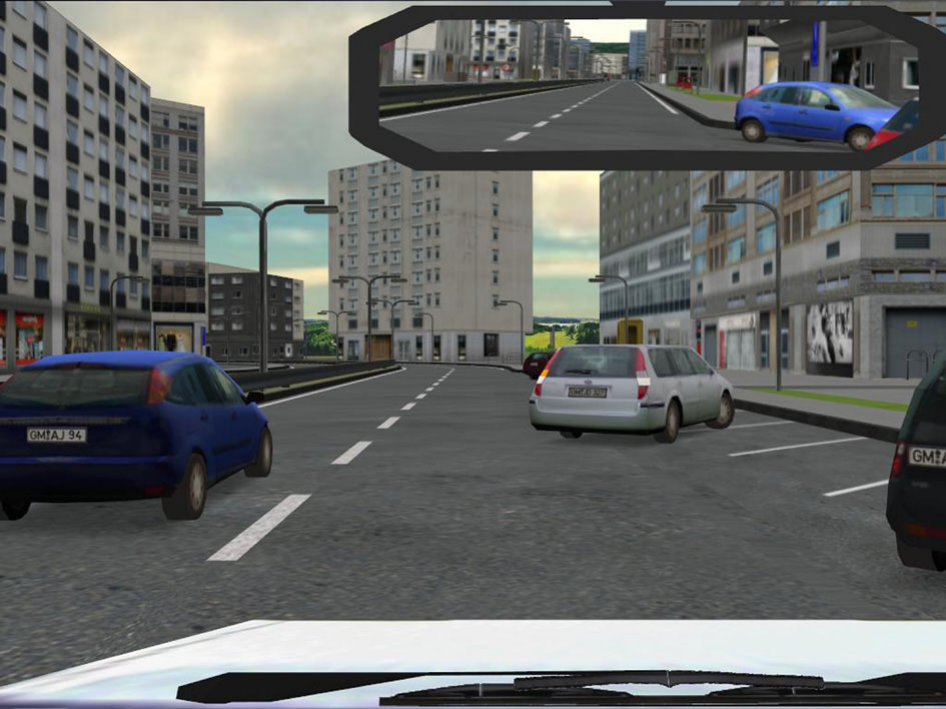
**Graphical illustration of a driving simulator scenario showing a town traffic situation (level 2, nice weather)**.

The goal of this training approach was to increase the mental workload of correct driving in a realistic multitasking driving setting. Therefore, complexity and difficulty were gradually increased from session to session. A training session took 40 min active driving and a short verbal feedback (feedback on reaction time, number of errors). Participants were instructed to drive with adequate speed and follow the instructions of the “simulated trainer.” The “simulated trainer” was a computer-based program: a male voice giving information about the direction of travel. These instructions were delivered according to Swiss traffic rules. The first training session included four scenarios (interurban, suburb, town, and motorway) without other vehicles in order to familiarize with the simulator. In the remaining nine training sessions six different traffic scenarios (interurban, suburb, town, motorway, overtaking and traffic rules scenario) with three different levels of difficulty were presented. Four or five scenarios were conducted in each training session (time duration of each scenario depends on the participants driving speed, no longer than 15 min for one scenario). Levels of difficulty were defined in each scenario with an increasing traffic frequency, increase of virtual drivers ignoring traffic rules (e.g., right of way rule) and an increase of hazardous traffic situations (e.g., child runs into the street). Additionally the complexity of traffic situation increased from interurban to suburban with highest complexity in the town scenario (see Figure 2). Furthermore, weather conditions were varied: in the third, sixth, and ninth training session it was raining infrequently or it was foggy. In training sessions four, seven, and ten participants had to drive in nighttime conditions. This training plan was fixed and participants had no possibility to adapt their subjective condition. As described above, one participant stopped due to excessive private demands.

The training progress was evaluated in four scenarios (three rural, one urban), which were not included in the training. In these scenarios, driving performance was measured using *driving errors* (accidents, traffic rule violation, leaving the lane, no use of direction indicator etc.), *top speed*, *mean speed*, *lane accuracy*, *lane variability*, and *reaction time* to unexpected stimuli (hazardous events). The driving simulator software automatically recorded the six variables for each of the four scenarios. These scenarios were conducted after the second, sixth, and tenth training session. The variables from the three rural scenarios were averaged. Training progress was analyzed with an ANOVA for repeated measures and showed a significant and positive effects, expect for the variable *lane accuracy* (*p* > 0.05). The detailed results are listed in Table 4.

**Table 4:** **Training progress: simulator training**.

**Variable**	**Second training session (***N*** = **31**)**	**Sixth training session (***N*** = **31**)**	**Tenth training session (***N*** = **31**)**		
	***M (SD)***	***M (SD)***	***M (SD)***	***F***	***p***
**RURAL SCENARIOS**					
Driving error	2.29 (1.47)	4.48 (2.79)	1.22 (1.41)	13.20	<0.01
Top speed	62.02 (5.82)	60.55 (5.30)	64.91 (4.87)	7.08	0.012
Mean speed	40.30 (3.88)	39.89 (3.44)	42.95 (3.34)	19.96	<0.01
Lane accuracy (%)	77.05 (4.50)	77.38 (4.71)	75.96 (4.70)	2.71	*n. s.*
Lane variability (%)	13.89 (1.47)	13.52 (1.16)	13.59 (1.14)	1.44	*n. s.*
Reaction time (s)	1.35 (0.13)	1.20 (0.11)	1.15 (0.14)	60.54	<0.01
**URBAN SCENARIO**					
Driving error	4.48 (2.79)	3.32 (2.31)	2.51 (1.99)	14.01	<0.01
Top speed	35.95 (4.69)	35.88 (5.03)	36.21 (4.62)	0.07	*n. s.*
Mean speed	16.33 (2.07)	17.07 (1.83)	17.80 (2.15)	16.99	<0.01
Lane accuracy (%)	65.42 (4.01)	65.95 (3.24)	65.65 (3.91)	0.69	*n. s.*
Lane variability (%)	25.58 (1.40)	24.88 (1.53)	24.80 (1.65)	7.73	<0.01
Reaction time (s)	1.11 (0.19)	1.15 (0.24)	1.05 (0.17)	1.77	*n. s.*

n. s., not significant; s, seconds.

### Attention training

The goal of this training approach was to increase specific driving-relevant cognitive functions. To prevent multitasking each scenario (intrinsic, phasic alertness, vigilance) was trained consecutively. A training session took 40 min active training and a short feedback (feedback on reaction time, number of errors and achieved level). The training regimes contained three different approaches. Each of the 10 training sessions was composed of 10 min intrinsic alertness training, followed by 10 min of phasic alertness training (Hauke et al., 2011) and 20 min of vigilance training, conducted by a software of Schuhfried GmbH (Schuhfried, n.d.). In all training sessions participants were seated on a chair.

In the two alertness training sessions, they saw a motorcycle from a driver's viewpoint, in motion. The motorcycle drove automatically a predefined circuit in a realistic driving scene. Speed and steering was controlled automatically by the software. Participants were instructed to react as fast as possible to objects and situations, which appeared during the ride. Objects were falling trees or rocks, cars, or animals crossing the street, and traffic lights changing to red. The visualized objects only require a reaction by pushing a corresponding button if they block the road. If participants reacted more than eight times fast enough (regularly stop to prevent a crash with an object) and/or made no further errors (e.g., anticipation), the software automatically increased the level of difficulty (e.g., increasing driving speed). In case of poorer performance during a training session level decreased.

In the vigilance training a virtual driver cabin's viewpoint was presented. The car drove automatically straight ahead with constant speed. Infrequently the car was overtaken. If the brake lights of the car now being in front light up, participants had to push a corresponding button as fast as possible. If participants did not push the button after 3 s, the brake light started to flash before an error was registered. The level of difficulty was controlled automatically by the software and increased after 15 CR with a reduction in overtaking maneuvers and reduction in surrounding visual stimulation (e.g., buildings, trees).

The training progress was evaluated for each session (reached training level) and was analyzed with an ANOVA for repeated measures. Participants in this training group showed a significant and positive training progress. The detailed results are listed in Table 5.

**Table 5:** **Training progress: cognitive training**.

**Variable**		**Friedman ***X*^2^** test (Comparison of levels from all training session)**	
	***N***	***X*^2^**	**Asymp. Sig**.
Intrinsic alertness	23	145.99	<0.01
Phasic alertness	23	157.05	<0.01
vigilance	23	185.95	<0.01

### Procedure

The general study design is a pre-post design. During the pre- and post-test sessions all participants conducted cognitive and on-road tests. Between the pre-test and post-test measurements the participants performed either the training regimes (driving simulator or attention) or simply waited to participate for the post-test (control group). Data acquisition took 25 months (May 2010–June 2012). The 91 participants were assigned to one of 13 training blocks. During every block seven participants took part (three for the simulator training, two for the attention training, and two for the control group). During the entire study, every participant took part in a single setting. Block duration was 7 weeks with two dates per week (two dates pre-tests; 10 training sessions; two dates post-tests; in total: 14 dates). In the first week (pre-test) and last week (post-test) on-road driving performance and cognitive performance was measured. Furthermore, all participants underwent electroencephalography (EEG) recordings during a set of three inhibition tasks (stroop, negative priming, and flanker). These data will be presented elsewhere (in preparation).

Before each computer test and on-road drive participants received an introduction about the test process and conditions (for tests: written instruction by software; for on-road drive: verbal instruction by the DI), but no feedback about their performance. Before all computerized tests (pre-test phase) the corresponding software automatically measured reaction time, correct and wrong answers to evaluate the participants' understanding of the particular test. In this pre-test phase participants were allowed to ask questions about the cognitive tests or in case of any other problems. During the first appointment participants conducted initially the cognitive test battery and thereafter the on-road drive (each lasting 1 h). On the second appointment (not included in this article), inhibition tasks (Stroop, negative Priming, Flanker) and EEG recording was conducted. From the second to the sixth week, both training groups participated in 10 training sessions with two training sessions a week. During this time period, the control group received no intervention. During post-test week on the second but last date, inhibition tasks and EEG recordings were conducted (not included in this article). On the last appointment, participants conducted again the cognitive test battery and thereafter the on-road drive (each lasting 1 h).

To control for mood and motivational changes during the training, participants completed in the first, fifth, and tenth training session an adapted version of the SAM (Self Assessment Manikin) for mood changes (Beeli et al., 2008) and an adapted version of the FAM (Fragebogen zur Erfassung aktueller Motivation) for motivational changes (Rheinberg et al., 2001). In the driving simulator training group, SS was measured by calculating the mean of the three main subjective symptoms: nausea (N), oculomotor (O), and disorientation (D) (Kennedy et al., 1993) at begin, middle and end of training. Each symptom was scored from 1 to 5 (low SS = 1, severe SS = 3, strong SS = 5).

### Statistical analysis

Statistics were calculated using SPSS 18 for Windows 7 (SPSS Inc., Chicago, Il.), with a significance level of α = 0.05. Differences in baseline performance and in demographic data between the groups were compared using Kruskal-Wallis and ANOVA tests.

With hierarchical multiple regression analysis with planned group comparisons the training benefits (dependent variables pre- and post-test) were analyzed for the cognitive and on-road performances. For the planned group comparisons orthogonal contrast coding was used. Contrast coding was used in accordance with the hypotheses formulated in the introduction.

We defined a-priori (planned) contrasts allowing us to test interaction effects (Pedhazur, 1982), which are of utmost importance to test our hypothesis formulated in the introduction. First we designed interaction contrasts allowing us to test pre-post differences between both training groups (attention and driving simulator training) vs. the control group. The second contrast was designed as orthogonal to the first contrast allowing us to test for pre-post differences between both training groups. Since we adopted orthogonal contrasts we only can use two contrasts (pre- and post-measures: *df* = 1; number of groups: *df* = 2).

The advantage of this contrast design is that we gain more statistical power to detect even moderately strong effects without increasing sample size too much. In addition, this kind of a priori defined testing is strongly hypothesis-driven. Since we anticipate that training results in improvement we decided to test uni-directionally. According to our hypothesis we are not interested to compare the two training groups separately with the control group since we are not interested in potential differences to the control group. We are mainly interested in differences between the training groups. We also focus statistical testing on the composite measure for on-road driving and cognitive performance. For the sub-measures of which the composite scores are calculated we only report the results on a descriptive basis.

Because of the relative small number of subjects and large number of dependent variables, which we can possibly be used for statistical testing, it is nearly impossible to perform classical statistical inference tests. The reason is the small power even when moderate or even strong effects are present. Thus, when applying corrections for multiple testing, no or only a few of strong effects would have been identified. Because of this, we decided to use a more descriptive statistical approach for most of the variables. For a subset of variables, we performed a strongly hypothesis-driven statistical analysis (the composite scores for on-road performance and cognitive test performance). For these tests we draw stronger conclusions from the analyses. For hypothesis-free analyses (the sub-measures constituting the composite scores), the statistical test results are not interpreted in terms of statistical significance, they are rather used as descriptive measures of between-group differences. For these analyses, we will be more reluctant in interpreting the findings. The *p*-values for these comparisons can be taken as measures of effect (Krauth, 1988). Since we have to consider the fact that *p*-values depend on sample size, we also calculate effect sizes according to Cohen (1988). A *d* > 0.3 and <0.5 is considered as small, a *d* > 0.5 and <0.8 as moderate, while a *d* > 0.8 is considered as large.

## Results

### Demographic and baseline group comparison

There were no differences between the groups with regard to daily driving activities, relevant demographic variables, or gender (all *p* > 0.05). Baseline comparisons showed a significant group difference in crystalline intelligence, decision time in the simple reaction task (RT) and district dependent behavior in the on-road assessment (Table 6). These variables did not correlate (as computed with Pearson correlations) with the composite scores for on-road and cognitive performance (all *p*-values at least < 0.10). In all other measures there were no significant baseline differences (all *p* > 0.05). Furthermore, no baseline differences exist for the overall on-road and cognitive performance.

**Table 6:** **Baseline differences between groups**.

**Variable**	**Simulator training group (***N*** = **31**)**	**Cognitive training group (***N*** = **23**)**	**Control group (***N*** = **23**)**	***p***
	***M (SD)***	***M (SD)***	***M (SD)***	
Crystallized IQ	125.6 (11.3)	128.9 (10.3)	118.6 (11.9)	0.009
Simple choice reaction (DS), ms	467.3 (81.5)	475.3 (90.7)	545.3 (91.2)	0.004
District dependent behavior	3.96 (0.78)	3.79 (0.77)	4.29 (0.47)	0.047

ms, milliseconds.

### Simulator sickness, emotional, and motivational status

Participants in the simulator training reported SS, which significantly changed during the training (*X*^2^ = 30.98, *p* < 0.001). Wilcoxon tests revealed a drop in subjectively experienced SS from the start of the driving simulator session (*median* = 2.17) to half time (*median* = 1.43, *z* = −4.21, *p* < 0.001) and at the end (*median* = 1.38, *z* = −3.83, *p* < 0.001) of the training. Therefore, no SS differences occurred between half time and end of training. Training groups differed in their emotional valence during training participation [*F*_(1, 52)_ = 4.56, *p* = 0.038]. *Post-hoc t*-test showed on average a lower positive valence in the driving simulator training group (*M* = 3.35, *SE* = 0.16) than in the attention training group at beginning (*M* = 4.43, *SE* = 0.16), [*t*_(52)_ = −4.67, *p* < 0.01]. No significant differences existed to half time and at the end of training. Furthermore, there was no group difference in emotional arousal and motivation (all *p* > 0.05).

### On-road training effect

Descriptive statistics from on-road performance are displayed in Table 7 including Cohen's *d* for the pre-post differences broken down for the three groups.

**Table 7:**
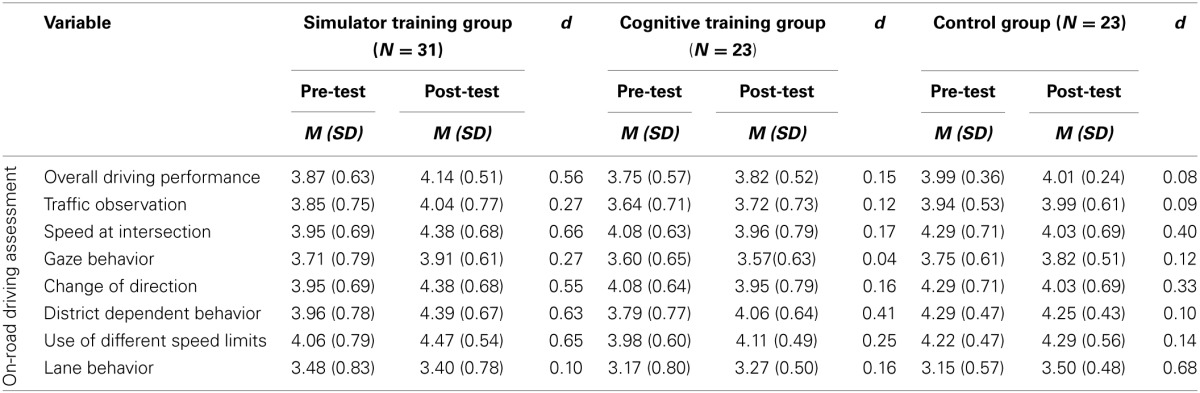
**Descriptive statistics for on-road measures**.

d, Cohen's d effect size (Cohen, 1988) using the pooled SD for both conditions and correcting for dependence between means according to Morris and DeShon (2002).

**Figure 3:**
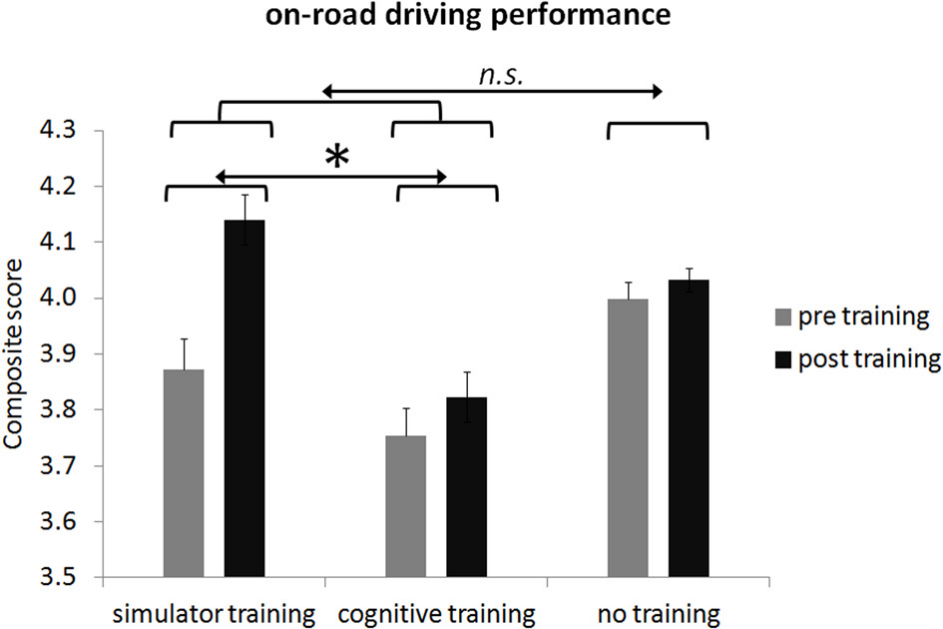
**Group means of overall on-road performance before and after participation broken down for the three groups**. Error bars in plots indicate the standard error of the mean. Please note dimension is arbitrary. *Note: n.s.*, not significant; ^*^ < 0.05.

Training gains and the regression model on overall on-road performance are displayed in Figure 3 and Table 8. Significantly different training gains for the different groups are displayed in Table 8. Compared to the control group, there was no significant change in the overall on-road performance [*F*_(1, 74)_ = 1.59, *p* = 0.11, *d* = 0.35] as a result of the training, but a significant improvement in the simulator training group compared to the attention training group [*F*_(1, 74)_ = 2.86, *p* < 0.05, *d* = 0.48].

**Table 8:** **Multiple regression for the interaction between orthogonal contrasts and training gain for the composite score of the on-road performance**.

**Variable**	***B***	***SE***	***β***
**Overall driving performance**			
Linear interaction AB × C	0.022	0.018	0.061
Linear interaction A × B	0.050	0.029	0.082^*^

A, driving simulator group; B, cognitive training group; C, control group. AB × C, comparison of the average of the training effect for group A and B vs. the training effect for group C.

A × B, comparison of the training effect for group A vs. the training effect for group B.

* < 0.05.

Please note that the following comparisons are only done on a descriptive basis to prevent an inflation of statistical tests. Increased performance for both training groups compared to the control group were found for the following sub-measures: *change of direction* [*t*_(74)_ = 2.24, *p* < 0.05, 1-tailed, *d* = 0.56], *district dependent behavior* compared to the control group [*t*_(74)_ = 2.62, *p* < 0.05, 1-tailed, *d* = 0.68]. Significantly better performance yielded for the simulator training group compared to the attention training group for the variable *change of direction* [*t*_(74)_ = 2.68, *p* < 0.01, 1-tailed, *d* = 0.79]. For *lane behavior* there was an increase in this measure for the control group compared to both training groups [*t*_(74)_ = −1.96, *p* < 0.05, 1-tailed, *d* = 0.54].

### Cognitive training effect

Descriptive statistics from cognitive performance are displayed in Table 9 including Cohen's *d* for the pre-post differences broken down for the three groups.

**Table 9:**
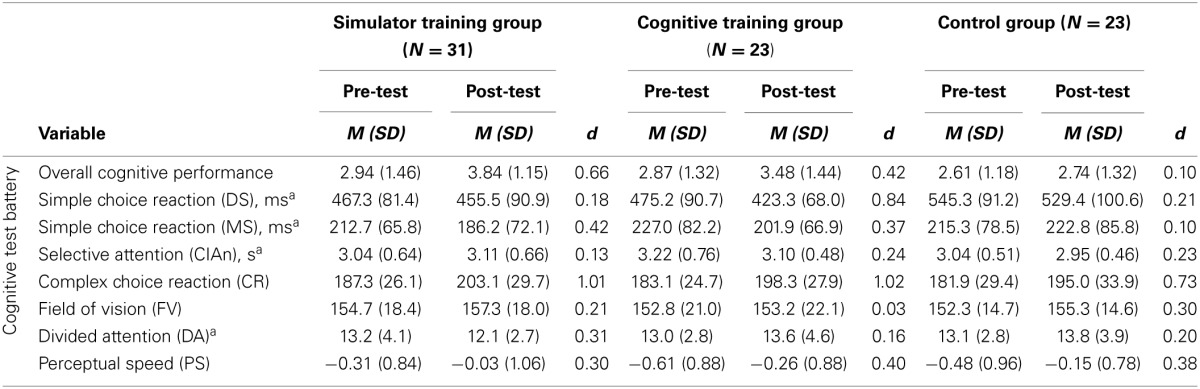
**Descriptive statistics for cognitive measures**.

^a^Smaller scores reflect better performance. RT, reaction time; s, seconds; ms, milliseconds; d, Cohen's d effect size (Cohen, 1988) using the pooled SD for both conditions and correcting for dependence between means according to Morris and DeShon (2002).

**Figure 4:**
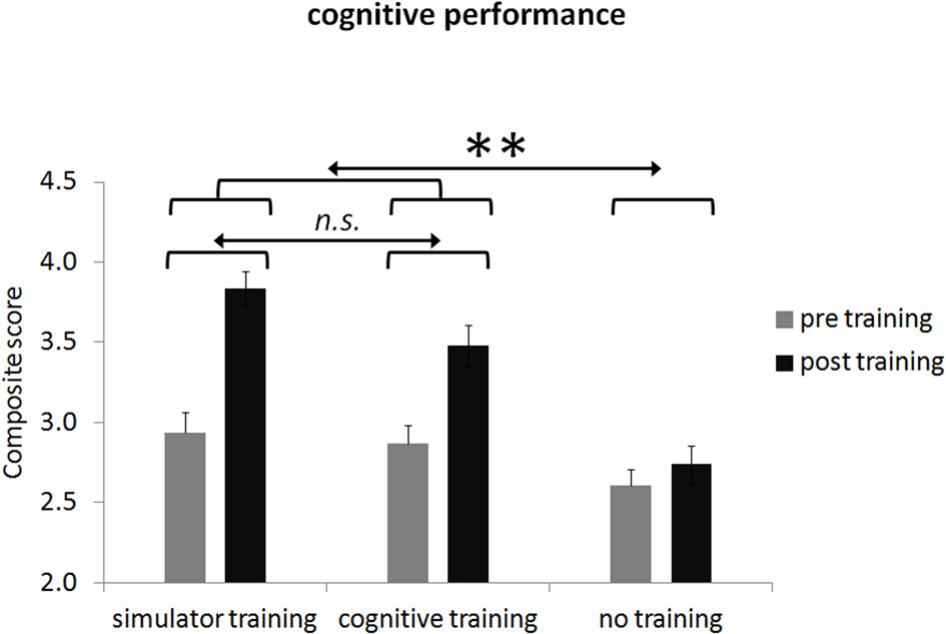
**Group means of overall cognitive performance before and after participation broken down for the three groups**. Error bars in plots indicate the standard error of the mean. Please note dimension is arbitrary. *Note: n.s.*, not significant; ^**^ < 0.01.

Training gains and the regression model on overall cognitive performance are displayed in Figure 4 and Table 10. Significantly different training gains for the different groups are displayed in Table 10. Compared to the control group, there was a significant improvement in the *overall cognitive performance* for both training groups [*F*_(1, 74)_ = 8.99, *p* < 0.01, *d* = 0.48] but no significant improvement in the simulator training group compared to the attention training group [*F*_(1, 74)_ = 0.36, *p* = 0.55, *d* = 0.22].

**Table 10:** **Multiple regression for the interaction between orthogonal contrasts and training gain for the composite score of the cognitive performance**.

**Variable**	***B***	***SE***	***β***
**Overall cognitive performance (NN)**			
Linear interaction AB × C	0.104	0.058	0.106^**^
Linear interaction A × B	0.074	0.096	0.045

A, driving simulator group; B, cognitive training group; C, control group. AB × C, comparison of the average of the training effect for group A and B vs. the training effect for group C.

A × B, comparison of the training effect for group A vs. the training effect for group B.

** < 0.01.

For the sub-measures of cognitive performance we revealed improved performance in several measures. As explained in the method section the following comparisons are only used on a descriptive basis to prevent an inflation of statistical tests. Increased performance for both training groups compared to the control group were found for the following sub-measures: *motor speed* compared to the control group [*t*_(74)_ = −1.98, *p* < 0.05, 1-tailed, *d* = 0.48]. Significantly better performance yielded for the attention training group compared to the simulator training group for the variable *decision speed* [*t*_(74)_ = −1.81, *p* < 0.05, 1-tailed, *d* = 0.56].

## Discussion

The main goal of this study was to investigate whether on-road driving in older healthy active drivers can be improved by two different training approaches: training with a driving simulator or training of cognitive functions known to be involved in controlling driving. Based on the current literature we hypothesized that both training approaches would increase on-road driving performance as well as cognitive performance. Secondly, we hypothesized that driving simulator training, in which scenarios are comparable to on-road driving, would induce stronger on-road driving performance gains than attention training because this training requires multitasking. We also anticipated that cognitive performance growth would strongly benefit from the simulator training since this training also induces lots of cognitive functions (e.g., attention, spatial perception, sensory-motor coordination and tracking, executive functions, vision, working memory etc.; Lees et al., 2010; Romoser, 2013; Casutt et al., 2014) and is a kind of multitasking training for which a recent paper has shown strong beneficial effects on cognitive performance and its underlying brain functions especially for older adults (Anguera et al., 2013). It is worth noting that we have used driving simulator training employing different naturalistic virtual realities with increasing complexity and difficulty. In addition, a well-established cognitive training software was used consisting of three consecutively conducted cognitive training approaches (Hauke et al., 2011).

Participants in the driving simulator training group improved their on-road driving performance compared to the attention training group. Cognitive performance, however, improved in both training groups (driving simulator and attention training groups) in comparison to the control group. Thus, the driving simulator group showed improvements in on-road driving performance as well as cognition (near and far transfer) while the attention training group only showed improvement in cognition (near transfer). Thus, the driving simulator training (as an example for a complex training) obviously induces near and far transfer and exerts stronger training gains what was suggested in previous influential reviews (Lustig et al., 2009; Zelinski, 2009).

But what are the reasons for the different learning effects on on-road performance and cognitive functions? Highly interactive and complex cognitive training approaches (not only using driving simulators but interactive video games) have been shown to exert positive influences on cognitive functions, behavior, emotion, and lots of other actions and functions (Green and Bavelier, 2006a,b; Achtman et al., 2008; Basak et al., 2008; Karbach and Kray, 2009; Marmeleira et al., 2009). In this context it has been argued that virtual environments and scenarios are inherently attractive and motivating (Lees et al., 2010). Many subjects feel a kind of presence when interacting with highly immersive video scenarios especially when they interact with or within the virtual environment (Havranek et al., 2012). These are circumstances enhancing attractiveness of these scenarios, which most likely also enhance motivation and attention, both factors, which are pivotal for learning and memory consolidation (Green and Bavelier, 2006a,b; Green et al., 2010; Bavelier et al., 2012). Therefore, it is most likely that attention and motivation to learn is stronger for those subjects who practice with a driving simulator than for those subjects who only practice more or less abstract cognitive functions. However, the rated motivation in our study did not differ between both training groups. Thus, it might be that the questionnaires measuring subjective motivation and arousal are not sensitive enough to capture fine graded motivation and arousal differences. It is well-known that subjective and physiological measures of motivation and arousal only weakly covariate (Erdmann and Janke, 1978). Thus, it is possible that our subjects participating in the driving simulator group were indeed stronger motivated or aroused (with the accompanying physiological changes) but without noticing it. In addition, it is also possible that all subjects were motivated or aroused to a quite high degree, which cannot be captured due to ceiling effects. Secondly, the driving simulator provides traffic scenarios, which are quite close and partly similar to real traffic situations. Thus, the subjects practicing with the driving simulator, train something, which they directly can use in real situations. Thus, the conceptual and practical “distance” of the learned aspects from a driving simulator context to an on-road driving situation is closer (near transfer) than the “distance” from attention training to on-road driving (far transfer). Similar beneficial effects from simulator training (even when the used simulators are simple) to real life actions have been demonstrated quite frequently for controlling specific problematic driving skills in older drivers (Romoser and Fisher, 2009; Lavallière et al., 2011, 2012; Romoser, 2013; Romoser et al., 2013). Even when the attention training is designed to be a bit more realistic and dynamic (e.g., visual search strategies at intersection) not only cognitive functions like DA, monitoring, and decision making improve but also on-road driving performance (Romoser and Fisher, 2009). Thus, the realistic and dynamic aspects of driving simulator training are most likely important factors enhancing learning and more importantly enhancing the improvement of on-road driving.

A further aspect of the driving simulator training might enhance improving on-road performance and cognition. Driving simulator training as we have used it in our study is very similar to multitasking training. During simulator driving the trainees have to orchestrate different psychological functions either simultaneously or in an elegant and efficient way sequentially. This kind of orchestration of several and different psychological functions is pivotal for efficiently driving a car. While driving in a driving simulator (and in a real car), the subjects have to control their car (sensorimotor control), scan the scenarios (perception), remember similar situations (memory), and anticipate as well as plan the maneuvers (cognition). Thus, this training has much in common with interactive cognitive multitasking (Basak et al., 2008; Marmeleira et al., 2009; Anguera et al., 2013). Moreover a recent publication showed that multitasking training increases not only performance in different cognitive domains (working memory, attention) but also induces changes in brain activity (Anguera et al., 2013). The authors interpreted their results of brain plasticity as an increased suppression of the default network during task engagement. In line with this evidence our results support the multitasking approach and its brain plasticity in the older adult brain and its positive transfer in cognition and on-road driving.

Additionally, in driving simulator studies it was shown that the level of multitasking costs is associated with driving uncertainty and driving errors (Bélanger et al., 2010) and that the multitasking costs in older drivers are greater than in younger drivers (Cantin et al., 2009). The multitasking nature of the driving simulator training is supported by the improved DA performance for the simulator group. DA is known to be a cognitive function relying on the complex interplay between different brain structures and is also a kind of multitasking. Cognitive training regimes during which one psychological function is practiced more or less isolated without switching lacks this dynamic interaction between different psychological functions (Zelinski, 2009).

Having a closer look at the improved aspects of on-road driving it becomes evident that they correspond to those traffic situations (behavior at crossroads, junctions, and lane change), which are discussed in the literature as typical problematic driving situations causing reduced driving safety and increased driving errors (Braitman et al., 2007; Romoser and Fisher, 2009; Lavallière et al., 2011). Romoser and Fisher (2009) showed that active simulator training improves older driver visual scanning strategies at intersections, which also were observed after a follow-up of 2 years (Romoser, 2013). Furthermore, these problematic driving behaviors are related to declines in executive functions, for example in decision making (Daigneault et al., 2002; Horswill et al., 2008; Romoser and Fisher, 2009). According to these results, the present study complements the existing research. Interactive and multitask simulator training increases higher order cognitive functions and everyday life abilities in older adults.

## Limitations

First of all it should be kept in mind that SS is still a problem at least for some subjects practicing with the driving simulator. However, reported average sickness diminished during the simulator training and even disappeared entirely for most of the subjects. Thus, between-groups differences with respect to these variables could not account for the improvement in on-road driving and cognitive performance. However, some subjects were excluded from the study when the sickness symptoms did not disappear or attenuate to a strong degree. Although only seven subjects were excluded because of SS, SS might have influenced the present results in several ways. For example, we measured those subjects who could cope with the sickness symptoms. Thus, their training performance might be linked somehow with this coping and struggling. Maybe they employ more self-control and/or self-discipline during training than those who didn't experience these obstacles.

There are also some baseline differences between the groups with respect to the driving performance and the performance in the cognitive tests, which are difficult to explain (e.g., district dependent behavior, or reaction times in some cognitive tests). However, since these baseline differences have only been identified in two measures and did not influence the overall on-road performance and overall cognitive performance we are sure that these differences do not influence training performance.

It should be noted that quality of lane behavior (a sub-variable contributing to on-road driving performance) did not improve as a consequence of the driving simulator training while the control group improved their performance with respect to this measure. This partly paradoxical finding is difficult to explain and we would like to refrain from making too strong and speculative arguments in this case. One tentative explanation could be that lane accuracy or its deviation is not a sensitive measure. In another simulator study comparing young and old drivers, there was no significant between-group difference with respect to this measure (Cantin et al., 2009). Further research is thus needed to study the moderating influences on this variable.

One important limitation in the present study is the absence of a further active control group to control for simple activity (even being unrelated to driving). Since this experiment was extremely demanding for the participating subjects (e.g., they had to travel to the psychological institute several times to practice the cognitive tasks or the driving simulator) it would have caused additional organizational effort to hire additional subjects for our active control group. In addition, it is borderline unethical to let a group of older adults practice something, which is unrelated to on-road driving and from which we anticipate no direct or indirect influence on on-road driving. We are thus sure that the local ethics committee never would have approved a control group like this. However, we used both experimental groups as control groups for the other group. Thus, the attention training group acted as control group for the simulator group and vice versa.

Training intensity and duration are also issues, which will have substantial impacts on training results, either for the attention or the driving simulator training. The training intensity and frequency used in our study might me too low to induce strong training gains. Thus, it would be interesting to study whether increased training durations and frequencies will result in stronger improvements in cognition and driving behavior.

Additionally, a critical point of our study is the specific sample of older adults. All subjects (irrespective to which group they have been assigned) were highly interested to participate and most of them were active drivers using their car frequently. For example, the average mileage in Switzerland for this age group is 3200 km (Bundesamt für Statistik and Bundesamt für Raumentwicklung, 2012). The mean mileage in the participating subjects varied between 8973 and 11,909 km. Whether subjects who are closer to the average mileage would benefit differently from the driving simulator or cognitive training has to be shown in different experiments. That the particular sample of older adults has an influence on driving improvement has been shown in a study of Roenker et al. (2003). They uncovered a positive influence of a cognitive training on on-road driving in high-risk older adults, which are deemed to perform suboptimal in real driving situations.

A final critical point is that during cognitive training not only cognition is improved but also other functions (e.g., perception). However, the exact nature of the relation between perception and cognition is currently unknown and has to be elucidated in future studies. (for a similar conjecture see Anstey et al., 2003). Thus, we are not in the position to delineate whether the subjects of our experimental group demonstrate improved sensory and perceptual functions as a consequence of our training approaches. However, we can state that cognitive functions are altered due to our training.

## Conclusion

In this study we directly compared the influence of attention training and simulator training on on-road performance and cognition. Here we showed that only participants practicing to drive in different traffic scenarios using a driving simulator significantly improved their on-road driving performance compared to a group involved in attention training. In addition, both training groups (the driving simulator and the attention training group) showed improved cognitive performance compared to a control group. Thus, the present study shows that driving simulators are useful training tools to improve on-road performance as well as cognition in older adults. Although this study supports the beneficial role of driving simulators to improve on-road driving (and cognition) further studies have to be conducted disentangling the different cognitive processes benefiting most from driving simulator training. In addition, it has to be shown how the measured on-road performance relates to those traffic measures, which are most important for real traffic like traffic safety or crash numbers. It will also be interesting how different samples of older drivers (e.g., at-risk drivers with mild or advanced cognitive problems) will benefit from driving simulator and/or attention training.

## Author contributions

Gianclaudio Casutt: Study conception and preparation, acquisition of data, statistical analysis, interpretation of data, drafting manuscript. Nathan Theill: Statistical analysis, interpretation of data, revising manuscript. Mike Martin: Revising manuscript. Martin Keller: Study conception. Lutz Jäncke: Supervision of study conception, statistical analysis, interpretation of data, revising manuscript.

### Conflict of interest statement

The authors declare that the research was conducted in the absence of any commercial or financial relationships that could be construed as a potential conflict of interest.

## Footnotes

^1^http://www.lafayetteevaluation.com/product_detail.asp?ItemID=353
